# Design and Performance Evaluation of a Distributed OFDMA-Based MAC Protocol for MANETs

**DOI:** 10.1155/2014/708798

**Published:** 2014-07-16

**Authors:** Jaesung Park, Jiyoung Chung, Hyungyu Lee, Jung-Ryun Lee

**Affiliations:** ^1^Department of Information Security, The University of Suwon, Suwon 445-743, Republic of Korea; ^2^School of Electrical and Electronics Engineering, Chung-Ang University, Seoul 156-756, Republic of Korea

## Abstract

In this paper, we propose a distributed MAC protocol for OFDMA-based wireless mobile ad hoc multihop networks, in which the resource reservation and data transmission procedures are operated in a distributed manner. A frame format is designed considering the characteristics of OFDMA that each node can transmit or receive data to or from multiple nodes simultaneously. Under this frame structure, we propose a distributed resource management method including network state estimation and resource reservation processes. We categorize five types of logical errors according to their root causes and show that two of the logical errors are inevitable while three of them are avoided under the proposed distributed MAC protocol. In addition, we provide a systematic method to determine the advertisement period of each node by presenting a clear relation between the accuracy of estimated network states and the signaling overhead. We evaluate the performance of the proposed protocol in respect of the reservation success rate and the success rate of data transmission. Since our method focuses on avoiding logical errors, it could be easily placed on top of the other resource allocation methods focusing on the physical layer issues of the resource management problem and interworked with them.

## 1. Introduction

The orthogonal frequency-division multiple access (OFDMA) has received attention as a promising air interface for next-generation wireless systems by providing high data rates while supporting good coverage and mobility [1]. OFDMA can simultaneously satisfy the communication requirements of multiple mobile stations by allocating one or more subcarriers to each mobile station at the same time unit (OFDMA symbol) [2]. Since OFDMA provides the flexibility in radio resource management, OFDMA has been adopted in many infrastructure based wireless networks such as IEEE 802.16 [3] and 3GPP LTE-A [4]. On the other hand, the wireless mobile ad hoc multihop communication paradigm is also receiving attention as a solution, not only for extending the coverage of wireless communications, but also for enhancing the quality of communication services in shadow areas [5, 6]. However, unlike an infrastructure-based network, an ad hoc network is self-organized by participating nodes without any regulations of centralized control entities such as base stations (BSs) or access points (APs). Hence, nodes in an ad hoc network should contend for communication resources, which may result in collisions in resource allocations. The main reason of the collision is that multiple nodes try to use the same resources at the same time. Since OFDMA allows multiple simultaneous communications by allocating a portion of resources to different nodes at the same time, combining the two promising technologies (OFDMA and wireless mobile ad hoc networks) is expected to provide enhanced communication opportunities.

Relay network has been designed by standard bodies to exploit the OFDMA in mobile ad hoc networks [7, 8]. The purpose of these relay networks is to extend the coverage of a BS and to increase the overall system throughput. In this network, a relay node plays the roles of both a BS and a mobile station (MS). However, a distinguishing feature of an ad hoc network is that each node could be not only a data source and destination but also a data forwarder to assist other nodes. Since a relay network evolves from infrastructure-based networks, a relay method is designed to operate with backward compatibility towards existing systems. Thus, the frame format of the legacy cellular systems, which is strictly divided into an uplink (UL) part and a downlink (DL) part in the time or frequency domain, does not reflect the unique features of an ad hoc network [9, 10]. Furthermore, the relay standards are mainly designed for two-hop communications between an MS and a BS through a relay node and the radio resources are still controlled by a BS. Therefore, it is difficult for a relay network to support more than 2-hop communication and it is not well-suited for an ad hoc network.

In OFDMA, the smallest resource allocation unit is defined by both time and frequency which will be called a protocol data unit (PDU) bin hereafter. In such networks, since every node can be a transmitter and a receiver, there could be multiple resource contentions for a set of PDU bins among different nodes while other nodes are exchanging data. However, since mobile ad hoc networks are required to be self-organized and operate without a centralized coordinator, collisions may occur during resource reservation and data transmission processes. Therefore, the utilization of radio resources would deteriorate unless the resource contention process is orchestrated in a distributed manner by taking into account the characteristics of such networks. So, to increase the utilization of radio resources by fully exploiting the flexibility provided by OFDMA in a mobile ad hoc multihop network while providing enhanced communication experiences to mobile users, an efficient MAC protocol operating in a distributed manner is required.

There are a few proposals on resource management for an OFDMA-based MAC protocol in mobile ad hoc networks. The method proposed in [11] mainly focuses on allocating PDU bins to maximize system throughput without considering medium access control. A signal strength based MAC protocol is proposed to reduce the cochannel interference and signaling overheads [12]. Each node selects a PDU bin to send data according to the interference level in the corresponding receiver. In [13], a resource allocation and conflict correction algorithm for an ad hoc network in a disaster area is proposed by considering two-hop interferences. Under the assumption that the interference range is twice as large as the communication range, the authors propose a resource allocation method to maximize the spatial reuse of resources. The authors in [14] take a cross-layer approach to design a resource management method in OFDMA-based ad hoc networks. They integrate a MAC layer and a routing layer to maximize the network throughput. They allocate resources based on the received signal strength at a physical layer to avoid interference.

Since these proposals focus on the physical layer issues of the resource allocation problem, they could either minimize the cochannel interference or maximize the system throughput by making a node to select a PDU bin based on the signal measurement at a physical layer. However, since it is very difficult (even if not impossible) for all nodes in an ad hoc network to have global information on network states in real-time, multiple nodes could select the same PDU bin while the other nodes are sending and receiving data via this PDU bin. Therefore, the corresponding data transfer would result in a collision even if each node reserves a PDU bin successfully. In [15], the logical errors are identified in the name of a multichannel hidden terminal problem. However, they extended the IEEE 802.11 MAC to operate in a multichannel environment without considering the important characteristics of OFDMA (i.e., support of simultaneous data transmission or reception to or from multiple nodes). In [16, 17], optimization approaches are taken to solve the resource management problem. In [16], an optimization problem is defined to maximize the throughput of mesh routers in an OFDMA-based mesh backhaul network. They proposed three heuristic methods to solve the optimization problem. However, they formulated the problem in a restricted ad hoc network where each node plays only one of the roles of a source, destination, and a relay. In [17], a convex optimization problem is solved by an interior point method to obtain optimal data routes, subchannel schedules, and power allocations to maximize a weighted sum rate of data communicated over the network. The proposals taking an optimization approach deal with the maximization of long term average utility of a network without considering the packet-level dynamics. Therefore, they could be used to explain the average behavior of data transmissions across the network and be useful in network planning. However, further elaboration is needed when they are adopted to design an online resource allocation method.

In this paper, we propose a distributed OFDMA-based MAC protocol for wireless mobile ad hoc multihop networks focusing on the resource management strategy to avoid logical errors that could happen in a resource management process. Therefore, proposed MAC protocol could operate on top of the other resource allocation methods that mainly focused on the physical layer issues of the resource management problem. To the best of our knowledge, this is the first distributed OFDMA-based MAC protocol for mobile ad hoc multihop network in the sense that the proposed MAC protocol fully exploits the characteristics of OFDMA-based MAC (simultaneous transmission or reception to or from multiple users using just one transceiver) and operates in a fully distributed manner without limitation on maximum hop count. The contributions in this paper are summarized as follows.

- We design a frame format reflecting the characteristics of a mobile ad hoc multihop network in which every node could be a transmitter as well as a receiver. In contrast to relay networks, a frame is not divided into a UL part and a DL part because radio resources might be wasted if the division cannot adapt to asynchronous traffic loads. Instead, we divide a frame into a data part and a control part. In the data part, radio resources are divided into protocol data unit (PDU) bins, each of which could be used for sending or receiving data according to the roles of nodes. The PDU bins are managed in a distributed fashion to support the features of an ad hoc network. All the nodes play equal roles in managing the PDU bins with the information in the control part of a frame.
- Since the proposed distributed MAC protocol uses the information of 1-hop neighbors not to compromise the autonomous nature of an ad hoc network, there exist some logical errors. We identify the scenarios of the logical errors and divide them into five categories according to their root causes. We present a detailed radio resource management method to avoid the logical errors and it is shown that three types of the logical errors are avoided while two types of them are inevitable under the proposed MAC protocol.
- The information of 1-hop neighbors is periodically broadcasted on a contention basis. We analyze the effect of the advertisement period on the delay from when a node broadcasts a message to when all of its neighbors successfully receive this message and determine the optimal advertisement period minimizing this delay.

The rest of the paper is organized as follows. In Section 2, we present the frame format and the distributed radio resource management method is detailed in Section 3. After we evaluate the performance of the proposed method through simulation studies in Section 4, we conclude the paper with possible future research directions in Section 5.

## 2. Frame Structure

**Figure 1:**
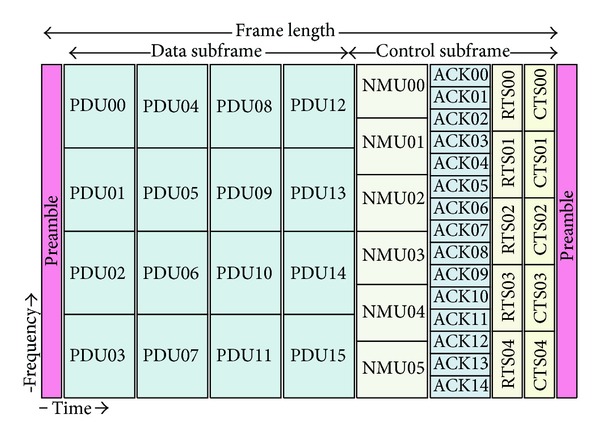
Example of frame structure (*N* = 16 PDU bins, *M* = 6 NMUs, *A* = 16 ACKs, and *B* = 5 RTS-CTS pairs).

In this section, we propose a MAC frame structure for a wireless mobile ad hoc multihop network reflecting the necessary distributed resource management procedures.  Figure 1 shows the structure of a MAC frame. A frame is divided into a data subframe and a control subframe with time. The data subframe is composed of *N* PDU bins. A PDU bin represents the minimum radio resource unit for data transmission and reception, which is made of *n*_*s*_ subcarriers and *n*_*t*_ OFDM symbols.

The control subframe is used for the nodes in a network to exchange control information so as to reserve a PDU bin in a distributed manner. According to the type of control information, the control subframe is further divided into a network management unit (NMU) zone, an acknowledgement (ACK) zone, and a read-to-send and clear-to-send (RCTS) zone. There are a number of channels in each zone separated by subcarriers. The number of channels in a zone is independent of those of the other zones, while the size and the function of the channels in the same zone are the same. The NMU zone is composed of *M* channels and is used for a node to announce its presence in an area. Each node periodically contends for a channel in the NMU zone to broadcast its presence to its 1-hop neighbors. By inspecting the NMU zone, a node could estimate its 1-hop neighbors in a distributed way.

Each channel in the ACK zone corresponds to each PDU bin. Thus, the number of channels in the ACK zone (*A*) is the same as the number of PDU bins (*N*). A channel in the ACK zone is used to control the data transmission and reception through the corresponding PDU bin. The usage of the ACK zone depends on the type of service request from an upper layer. If an upper layer requests a reliable data transfer service between adjacent nodes, a receiver sends an acknowledgement to a sender through the ACK channel when it receives data through the corresponding PDU bin. In contrast, if an upper layer requests a time-sensitive service to serve error-tolerant applications such as VoIP or video streaming, a channel in the ACK zone is used to control the data flow between a sender and a receiver. For example, a receiver may use the channel to feed back a message for flow control or to report the quality parameters of data reception such as the average delay and the data error rate [18–20].

When a node needs to send data, it uses the RCTS zone to reserve a PDU bin. The RCTS zone is further divided into an RTS region and a CTS region with time. Since these channels are used to convey small control frames for resource reservation, there could be *B* such pair of channels at the same time. The rationale behind having multiple simultaneous RTS-CTS pairs is to support the characteristics of mobile ad hoc multihop networks, where multiple reservations could be made at the same time. An RTS channel is coupled with a CTS channel. A pair of nodes reserves a PDU bin by exchanging an RTS frame and a CTS frame through a pair of RTS and CTS channels. For example, if a node trying to reserve a PDU bin sends an RTS frame to a receiver through the *k*th RTS channel, the receiver must respond by sending a CTS frame via the *k*th CTS channel.

A node could use multiple PDU bins for data transmission and reception if the PDU bins are separated in time. However, since we assume that a node has a single radio interface, a node cannot transmit while it is receiving and vice versa. Accordingly, a node cannot use PDU bins with the same OFDM symbols for both data transmission and reception at the same time. For example, a node cannot receive anything through any of PDU bin 04, PDU bin 06, and PDU bin 07, while it is transmitting data using PDU bin 05.

## 3. Operational Procedures

In this section, we propose a distributed radio resource management method using the MAC frame structure introduced in Section 2. The resource management method is composed of a network state estimation process and a resource reservation process. The network state estimation process operates in a management plane whenever a node receives a frame. The process is used for a node to estimate a set of 1-hop neighbor nodes and a set of PDU bins being used by them. The resource reservation process operates in a control plane to reserve a PDU bin when a node has data to send. The notations we use hereafter are summarized in Notations section.

### 3.1. Network State Estimation

**Figure 2:**
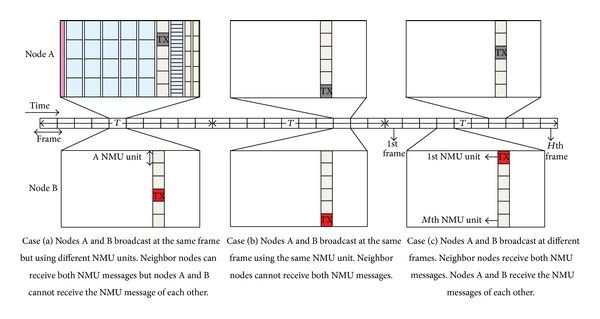
During time period *T*, each node selects a frame randomly among the frames within *T* and chooses an NMU channel in the frame at random to broadcast its presence to its 1-hop neighbors.

Each node advertises its presence to its 1-hop neighbors using a channel in the NMU zone. Since *M* channels in the NMU zone are shared by all the nodes, a collision may occur if more than two nodes in the transmission range of each other select the same NMU channel of the same frame at the same time. In addition to the randomness in selecting an NMU channel in a frame, we introduce additional randomness to reduce the collision probability. As shown in Figure 2, during every time period *T*, a node randomly selects a frame among the frames within *T* and chooses an NMU channel in the selected frame at random. Whenever a node receives a frame, it keeps updating NN_*X*_ by analyzing the NMU zone of the frame.

A node might manage *U*_*X*_ by examining the data subframe whenever it receives a frame. However, an exposed node problem may occur if a node estimates *U*_*X*_ by analyzing the data subframe. Since each ACK channel is coupled with its corresponding PDU bin and data frames collide at a receiving node, we take an approach that involves a node managing *U*_*X*_ by analyzing the ACK zone of each frame it receives.

A node *X* successfully receives an NMU message sent by *Y* in AN_*X*_ only if the other neighboring nodes in AN_*X*_ do not use the same NMU channel in the same frame selected by *Y* and *X* is in the receiving mode. Therefore, the probability that *X* successfully receives an NMU message sent by *Y* is derived as follows. The probability that *X* is in the receiving mode when *Y* is sending an NMU message is *P*_1_ = (*T* − 1)/*T* and the probability that none of the neighbors of *X* except *Y* sends an NMU message at the same time when *Y* broadcasts its NMU message also becomes *P*_1_. In addition, the probability that *Z* is in AN_*X*_ (*Z* ≠ *Y*) and *Y* uses different NMU channels even if *Z* and *Y* simultaneously send their NMU messages becomes *P*_2_ = 1/*T* × (*M* − 1)/*M*. Therefore, the probability that *X* successfully receives the NMU message sent by *Y* is given as

(1) pNMU=P1(P1+P2)|ANx|−1=T−1T(T−1T+1T(M−1M))|ANx|−1.

Since the frame length is *T*_*F*_, the average delay that *X* receives an NMU message from *Y* becomes

(2) $$\begin{array}{c} D_{o}=T_{F}\cdot T\overset{\infty }{\underset{k=0}{\sum }}\left(k+1\right){\left(1-p_{\text{NMU}}\right)}^{k}p_{\text{NMU}}=\frac{T_{F}\cdot T}{p_{\text{NMU}}}\;\left(\text{ms}\right). \end{array}$$

From (1) and (2), we can derive the optimal period T* that minimizes *D*_*o*_ by solving *dD*_*o*_/*dT* = 0 as

(3) $$\begin{array}{c} T^{*}=\frac{2-\left|{\text{AN}}_{x}\right|\left(a-1\right)+\sqrt{{\left(\left|{\text{AN}}_{x}\right|\left(a-1\right)\right)}^{2}+4a}}{2}, \end{array}$$

where *a* = (*M* − 1)/*M*. Therefore, if the number of NMU channels and the number of neighboring nodes are given, each node can determine the optimal period T*.

To construct an exact NN_*X*_, a node *X* must receive all the NMU messages from all of its neighboring nodes. Since a node could move around, it becomes important to know the average delay until *X* makes up an exact NN_*X*_. The probability that *X* receives an NMU message from *Y* in AN_*X*_ at least once before the *k*th period is given as *p*_*a*_ = 1 − (1−*p*_NMU_)^*k*^. Thus, the probability that *X* receives all the NUM messages from all the nodes in AN_*X*_ before the *k*th period is given as *P*_*b*_(*k*) = *p*_*a*_^|AN_*x*_|^. Therefore, the average delay that a node receives NMU messages from all of its neighbors becomes

(4) $$\begin{array}{c} D_{a}=\overset{\infty }{\underset{k=1}{\sum }}\frac{kdP_{b}\left(k\right)}{dk}‍. \end{array}$$

### 3.2. Distributed Resource Reservation Process

A node with data to send starts a resource reservation process by selecting a PDU bin to reserve. Among the *N* − |*U*_*X*_| available PDU bins, a node *X* randomly chooses a PDU bin *i* (i.e., *i* ∉ *U*_*X*_), where |*U*_*X*_| denotes the cardinality of the set *U*_*X*_. Then, the node *X* selects an RTS channel *j* at random among the unused RTS channels and sends *R*_*i*_^*j*^ to a receiver node *Y*. When *Y* successfully receives *R*_*i*_^*j*^ from *X* and no possible errors are detected, it answers with *C*_*i*_^*j*^, notifying *X* of successful reservation of the PDU bin *i*. If *X* successfully receives *C*_*i*_^*j*^ from *Y*, the PDU bin *i* is reserved between *X* and *Y*. After the reservation, *X* sends data to *Y* through the PDU bin *i*. When *Y* receives data without an error, *Y* sends an acknowledgement using the ACK channel *i* that corresponds to the PDU bin *i* if an upper layer requests a reliable data transfer service. If an error is reported, the sender *X* immediately retransmits the data frame using the same PDU bin used before. After successful data transmission between the sender and the receiver, the reserved PDU bin is returned. However, if the size of data is larger than the size of the PDU bin, the PDU bin is preempted. In other words, the sender sends data consecutively through the reserved PDU bin without additional exchange of RTS/CTS frames.

However, since each node uses only the local information estimated from the control subframes to reserve a PDU bin, the uncertainty in the estimated network state might fail a resource reservation attempt between two nodes. The failures can be classified into two categories. The first category of the failures represents the cases where a control message sent from a node collides with the other control messages sent from the other nodes. We will call this type of failure as a physical error. In contrast, the second category of the failures occurs when a reserved PDU bin leads to the failure of data transfer, even if the PDU bin is reserved in advance between two nodes through successful RTS/CTS frame exchange. We will call this type of failure a logical error. When a physical error occurs, our protocol operates as follows.

*(i) RTS Frame Collision.* If *Z* ∈ NN_*Y*_ sends an RTS frame using the RTS channel *j* when *X* sends an RTS frame to *Y* through the same RTS channel *j*, the two RTS frames collide with each other at *Y*. Since a node cannot receive a frame while it is sending, *X* cannot detect the collision, even if *Z* ∈ NN_*X*_. However, when the RTS frame from *X* collides, *Y* cannot respond with a CTS frame. If *X* does not receive a CTS frame from *Y* at the following CTS zone, it considers that the previous reservation request has failed. When *X* detects the RTS collision, *X* retransmits an RTS frame with the probability *p*_*rt*_ = 1/(NN_*X*_ + 1) so as to avoid successive collisions.

*(ii) CTS Frame Collision.* If *Z* ∈ NN_*X*_ sends a CTS frame through the CTS channel *j* when *Y* sends a CTS frame to *X* through the same CTS channel *j*, the two CTS frames collide with each other at *X*. Since *X* is in the listening mode, *X* could detect the collision of the CTS frames. After detecting the collision, *X* restarts the reservation process with probability *p*_*rt*_.

The logical errors occur because multiple node pairs could begin their resource reservation processes at the same time, while the other nodes are sending and receiving data. Since a logical error means that data transmission with a reserved PDU bin inevitably fails, network resources are wasted once a logical error takes place. However, since the uncertainties in estimating network states cannot be eliminated completely, we try to avoid logical errors in the resource reservation process by checking the RTS region of the frame once a node receives an RTS frame. The rationale behind this operation is that a node requesting a reservation does not know the resource usage situations of 1-hop neighbors of its receiver and data collides only at a receiver node. In Table 1, we classify the types of logical errors according to the situations in which they occur and describe how our protocol operates when it detects them. When a node detects a logical error, it begins another reservation process without random backoff to expedite the process. In the following, we explain the operational procedures for detecting and treating each type of logical error in detail.

*(iii) Logical Error Type 1 (LET1).* When a node *X* sends an RTS frame to *Y*, it selects a PDU bin *i* randomly among those that are not in *U*_*X*_. Therefore, a logical error type 1 occurs if *i* is in *U*_*Y*_ and a node in NN_*Y*_ that reserved the PDU bin *i* keeps using the PDU bin *i* when *X* sends data to *Y* through the same PDU bin *i*. Since *Y* cannot know how long the PDU bin *i* will be occupied, we take a conservative approach to avoid this type of logical error. When *Y* receives a request to reserve a PDU bin *i* from *X*, *Y* checks whether or not PDU bin *i* is in *U*_*Y*_. If *i* ∈ *U*_*Y*_, the data transmission from *X* through PDU bin *i* might collide with the other data transmission by a node in NN_*Y*_ at *Y*, even if *X* and *Y* reserve PDU bin *i* successfully. Thus, *Y* informs *X* of an LET1 by sending a CTS frame to *X* if the PDU bin *i* is in *U*_*Y*_. When *X* receives an LET1 from *Y*, *X* starts the resource reservation process again by selecting another available PDU bin *j* randomly.

**Figure 3:**
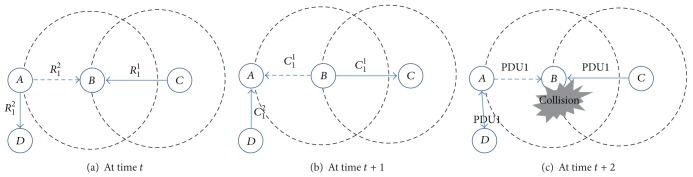
An example of operational procedure for a node to detect and treat an LET2. (In the following figures, we use frame time units. A circle represents the transmission range of a node located at the center of the circle. The solid arrows represent the intended direction of a frame and the dotted arrows represent the propagation of a frame to the nodes that are not the destinations of the frame. PDU*x* denotes data sent through PDU bin *x*.)

*(iv) Logical Error Type 2 (LET2).* When multiple adjacent node pairs try to reserve the same PDU bin using different RTS channels at the same time, a logical error type 2 may occur. We illustrate an example scenario in Figure 3. Since *A* and *C* are two-hop neighbors to each other, they could select the same PDU bin 1 when they begin their resource reservation process.  Figure 3(a) shows a situation where a node *A* is sending *R*_1_^2^ to a node *D*, while a node *C* is sending *R*_1_^1^ to a node *B* to reserve the same PDU bin 1 at the same time. The RTS frame sent from *A* to *D* also reaches *B*. However, since the RTS channel used for *A* to send the RTS frame to *D* is different from the one that *C* used to send its RTS frame to *B*, the two RTS frames do not collide at *B*. If PDU bin *i* is neither in *U*_*B*_ nor in *U*_*D*_, *D* and *B* send *C*_1_^2^ and *C*_1_^1^ to *A* and *C*, respectively, to inform *A* and *C* of the successful resource reservation at time *t* + 1, respectively (Figure 3(b)). However, the data sent from *A* to *D* also reaches *B*, which makes the data transmission from *C* to *D* fail, because the two data arrive at *B* through the same PDU bin at the same time (Figure 3(c)). To detect this type of logical error, if a node receives a request to reserve a PDU bin from one of its neighbors, it checks not only *U*_*X*_, but also *A*_*X*_, by investigating the RTS region of the frame from which it receives a resource reservation request. If a node detects an LET2, it sends a CTS frame to the node requesting a resource reservation with an error code LET2. When a node receives a CTS frame with LET2, it begins another resource reservation process. For example, in Figure 3(b), since the PDU bin that *A* (*A* ∈ NN_*B*_∧*A* ≠ *C*) attempts to reserve with *D* is in *A*_*B*_, *B* perceives an LET2. Then, *B* informs *C* of LET2 by sending a CTS frame with an error code LET2. *C* restarts the resource reservation process by selecting another PDU bin *j* randomly so that *j* ∉ *U*_*C*_∧*j* ≠ *i*.

**Figure 4:**
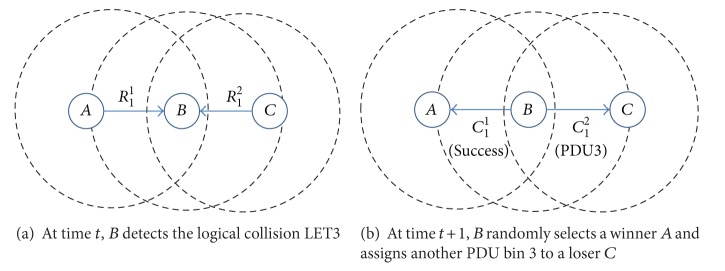
An example of operational procedure to detect and treat an LET3. (When *B* detects an LET3, *B* randomly selects a winner and informs the winner of the successful reservation. *B* sends a *C*_1_^2^ to *C* with another PDU bin 3 that is not in *U*_*B*_.)

*(v) Logical Error Type 3 (LET3).* When multiple nodes are sending their RTS frames to the same node through different RTS channels at the same time, a logical error type 3 occurs. In Figure 4, we show an example where an LET3 takes place. Since nodes *A*, *B*, and *C* are located such that *A* and *C* are members of NN_*B*_, *A* ∉ NN_*C*_, and *C* is not in NN_*A*_, *A* does not know the resource usage situation around *C*, nor does *C* know *U*_*A*_. If *A* sends *R*_1_^1^ to *B* while *C* is requesting *B* to reserve the same PDU bin 1 by sending *R*_1_^2^, the two RTS frames do not collide at *B*. Since *B* checks the RTS region of the received frame, *B* can detect that both *A* and *C* are trying to reserve a PDU bin 1. In this case, *B* could send RTS frames with an error code LET3 to fail both of the reservation requests. However, to reduce the overhead incurred by repeated resource reservation attempts, *B* randomly selects a winner. If *A* is selected as a winner, *B* sends *C*_1_^1^ to *A* to inform that the PDU bin 1 has successfully been reserved. On the other hand, *B* may send *C*_1_^2^ to *C* with an error code LET3 for *C* to restart the reservation process. However, to further reduce the overhead of repeated reservation attempts, in our protocol, *B* sends *C*_1_^2^ to *C* with a new PDU bin 3 that is neither in *U*_*B*_ nor in the RTS region of a frame received at time *t* (Figure 4(b)). If *C* receives a CTS frame containing a PDU bin that is not the same as the one it requested, *C* detects an LET3. Then, *C* uses the assigned PDU bin 3 if it is not in *U*_*C*_. Otherwise, it restarts the resource reservation process with another randomly selected PDU bin.

### 3.3. Inevitable Resource Reservation Errors

In our protocol, when a node *X* receives a resource reservation request, it investigates *U*_*X*_ and *A*_*X*_ to detect a logical error. Each node manages the local information (*U*_*X*_ and *A*_*X*_) by checking the control subframe of every frame that it receives. However, since each node competes for the NUM zone and the RCTS zone in a control subframe, estimation errors are involved in *U*_*X*_ and *A*_*X*_. In addition, a node cannot know the resource usage situation of its 2-hop neighbors. Therefore, there might be logical errors that could not be detected using only the information of 1-hop neighbors. According to the causes of errors, these logical errors can be further divided into two types. The first one is called a logical error type 4 (LET4) and is attributed to the fact that the *U*_*X*_ and the *A*_*X*_ might not exactly reflect the actual resource usage states of 1-hop neighbors all the time. The second one is denoted by a logical error type 5 (LET5) and takes place because a node does not know the states of its 2-hop networks.

**Figure 5:**
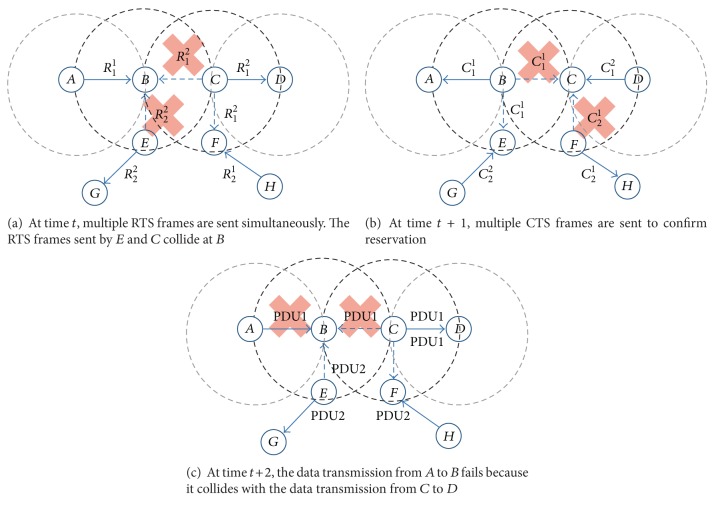
An LET4 takes place because local information cannot reflect the network state completely.

In Figure 5, we illustrate a situation where an LET4 occurs. At time *t*, *A* sends *R*_1_^1^ to *B*, *C* sends *R*_1_^2^ to *D*, *E* sends *R*_2_^2^ to *G*, and *H* sends *R*_2_^1^ to *F* to start the resource reservation processes simultaneously (Figure 5(a)). After receiving an RTS frame from *A*, *B* first checks *U*_*B*_ to detect an LET1. If the PDU bin 1 requested by *A* is not in *U*_*B*_, *B* checks *A*_*B*_ to detect LET2 and LET3. At time *t*, the RTS frames sent by *C* and *E* also reach *B*, even if they are destined to 2-hop neighbors of *B*. However, since the two frames are sent through the same RTS channel 2, they collide at *B*. Therefore, *B* cannot know that its neighbor *C* is attempting to reserve the same PDU bin 1 that *A* requested. In other words, *B* makes a mistake that the PDU bin 1 is not in *A*_*B*_ and sends a CTS frame *C*_1_^1^ to *A* to confirm the reservation. While the CTS frame *C*_1_^1^ sent from *B* is arriving at *A*, nodes *D*, *F*, and *G* also send CTS frames to their corresponding nodes at the same time *t* + 1. In this case, the CTS frames sent by *B* and *F* collide at *C* because they use the same CTS channel. However, since the CTS frames are not destined to *C*, all the reservation attempts succeed at time *t* + 1 (Figure 5(b)). Consequently, at time *t* + 2, nodes *A*, *C*, *E*, and *H* send their data using the reserved PDU bins. Since nodes *C* and *E* are in NN_*B*_, not only the data sent by *A* but also the data sent by *C* and *E* also arrive at *B*. Accordingly, the data sent from *A* to *B* collides with the data sent from *C* to *D* because *A* and *C* reserved the same PDU bin 1 (Figure 5(c)). As a result, the data transmission from *A* to *B* fails, even if they reserved a PDU bin successfully.

In our protocol, when a node *X* detects an LET3, it randomly selects a winner and notifies the winner that a resource reservation request has succeeded. The node also sends a CTS frame to the loser with another PDU bin that is different from the one requested by the loser. Instead of failing all the nodes, causing an LET3, our design choice might increase the success rate of a PDU bin reservation and decrease the singling overhead in a resource reservation process. However, such an operation might bring about another type of logical error called an LET5, because a node cannot know the resource usage states of its 2-hop neighbors.

**Figure 6:**
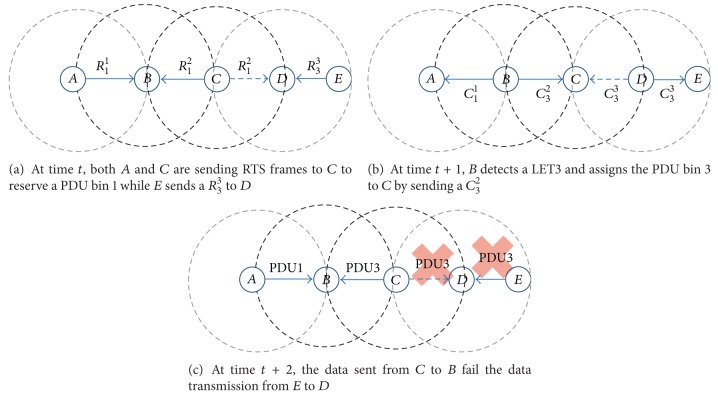
An example scenario of an LET5.

Figure 6 shows an example scenario of an LET5. At time *t*, both *A* and *C* are sending RTS frames to *C* to reserve a PDU bin 1 while *E* sends *R*_3_^3^ to *D* (Figure 6(a)). Since *A* is using an RTS channel 1 while *C* is sending an RTS frame through an RTS channel 2, the two RTS frames do not collide at *B* but cause an LET3. At time *t* + 1, *D* confirms the resource reservation request by sending *C*_3_^3^ to *E*. In contrast, *B* detects an LET3, because *B* perceives that more than two nodes are asking it to reserve the same PDU bin at the same time. If *A* is randomly selected as a winner, *B* sends *C*_1_^1^ to notify *A* of the successful reservation of the PDU bin 1. In addition, *B* randomly selects a PDU bin 3 that is neither in *U*_*B*_ nor in *A*_*B*_ and sends *C*_3_^2^ to *C* to expedite the resource reservation process of *C* (Figure 6(b)). When *C* receives *C*_3_^2^, it detects an LET3 and checks whether or not the assigned PDU bin 3 is in *U*_*C*_. Since *C* manages *U*_*C*_ by analyzing the ACK zone of a frame, the PDU bin 3 is not in *U*_*C*_, even though *D* is in NN_*C*_ and reserves the PDU bin 3 at time *t* + 1. As a result, *C* sends data to *B* using the PDU bin 3 at time *t* + 2. However, since the data also reaches *D*, the data transmission from *E* to *D* fails, even though they use the reserved PDU bin (Figure 6(c)).

## 4. Performance Evaluation and Discussion 

In this section, we evaluate the performance of our MAC protocol by analyzing simulation results in a variety of operational environments. Here, it is noted that the performance of the proposed MAC protocol is not compared to those of previous OFDMA-based MAC protocols since there are no protocols that conform to the basic operational principles of our MAC protocol (simultaneous transmission or reception to or from multiple users using just one transceiver, fully distributed scheduling, and no limitation on maximum possible hop count).

We evaluate the performance of the proposed resource management method with two performance metrics. The first metric is the reservation success rate (*Cr*) defined as the ratio of the number of CTS frames successfully received to the number of RTS frames sent including retransmissions. The second metric is the success rate of data transmission (*Dr*), which is defined as the proportion of the number of successful data receptions to the number of data transmissions including retransmissions. Regarding the MAC frame format for the simulation studies, we use the frame structure presented in Figure 1. There are 16 PDU bins in the data subframe. Accordingly, the number of channels in the ACK zone is set to 16. The number of NMU channels is configured to be 6 and there are 5 pairs of the RTS/CTS channels. Assuming a unit disk model, we set the same transmission radius of 2 km for all the nodes. We uniformly deploy nodes in a 16 km × 16 km area. The performance of our resource reservation protocol is influenced not only by the amount of traffic generated per node but also by the density of nodes. In this simulation topology, we use *ρ* to denote the number of nodes in a 4 km × 4 km region (the density of nodes) and we vary *ρ* from 2 to 12.

In a multihop network, the amount of data that a node transmits is the sum of a volume of data generated and an amount of data forwarded for its neighbors. The latter will vary according to the routing protocol used to determine the next hop of a flow, even if the operational environment of a network is the same. However, the focus of this section is to evaluate the performance of our resource reservation protocol regardless of other protocols. Thus, to exclude the influence of a routing protocol, we set up simulation scenarios in which a node transmits only the data it generated without forwarding data received from its neighbors. Data in a node is generated as follows. The data generation rate of a node follows a Poisson distribution with mean *λ* and the size of data follows an exponential distribution with mean *μ*. By varying *λ* and *μ*, we control the amount of data produced by a node. We further assume that one PDU bin is reserved for data transmission between a sender and a receiver. Once a reserved PDU bin is used to transmit data, it is returned, and a node should contend for a PDU bin again to transmit more data. However, if the size of data is larger than the size of a PDU bin, the data is segmented to fit into the PDU bin. Once a PDU bin is reserved for the first segment, the rest of the segments are consecutively transmitted through the PDU bin without additional resource reservation. In addition, if an error occurs while transmitting data, a node retransmits the data immediately through the same PDU bin reserved before without going through a resource reservation process again. We limit the number of retransmissions to 2. Thus, if data transmission fails after retransmitting twice, the data is discarded.

### 4.1. MAC Level Performance

In this section, we evaluate the performance of our distributed resource reservation protocol at the MAC layer in a static environment, where nodes do not move around and frames are not lost in a wireless link. This ideal operational environment is configured to evaluate the pure performance of our resource reservation protocol. In this environment, we measure the *Cr* and the *Dr* after all the nodes identify their 1-hop neighbors exactly to exclude the effect of the network state estimation process. In addition, in this operational environment, data transmissions fail only when collision occurs in a resource reservation stage or a data transmission stage.

**Figure 7:**
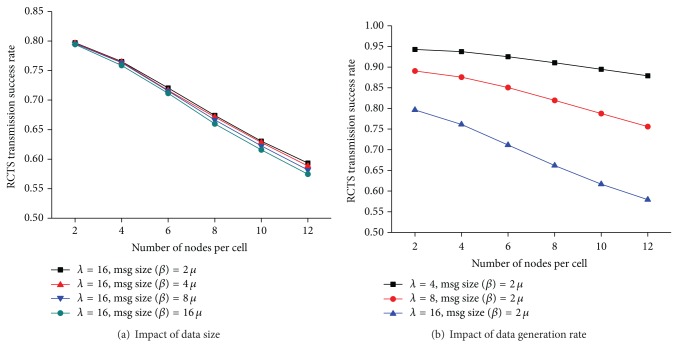
Resource reservation success rate at the MAC layer.

Figure 7 shows the reservation success rate for different node densities, data generation rates, and data sizes. A resource reservation process fails when a physical error takes place. A physical error occurs when an RTS frame or a CTS frame collides. A node *Y* cannot successfully receive an RTS frame *R*_*i*_^*j*^ sent from *X* if more than two nodes in NN_*Y*_ send *R*_*k*_^*j*^s while *Y* is receiving *R*_*i*_^*j*^. Similarly, a node *X* cannot decode a CTS frame *C*_*i*_^*j*^ sent from *Y* if more than two nodes in NN_*X*_ send *C*_*k*_^*j*^s while *X* is receiving *R*_*i*_^*j*^.

As the number of nodes in a network increases, it becomes more likely that more than two neighboring nodes are sending RTS frames or CTS frames using the same RCTS channel at the same time. Therefore, Cr decreases with the density of nodes. In our protocol, once a node reserves a PDU bin, the node uses the PDU bin consecutively if the size of data is larger than that of a PDU bin. Therefore, a node holds the reserved PDU bin longer as the size of data increases. Given the same operational environment, the average number of reserved PDU bins increases with the data size. If a node with data to send senses that a PDU bin is not available (i.e., *N* = |*U*_*X*_|), the node defers data transmission until it becomes *N* > |*U*_*X*_|. As the number of nodes waiting to start a reservation process becomes larger, the number of nodes beginning to send RTS frames simultaneously increases, which leads to the relative decrease in *Cr* with *μ* (Figure 7(a)). On the other hand, the number of data a node has to transmit increases with *λ*. Since a node has to reserve a PDU bin before it transmits data, the number of nodes attempting to reserve PDU bins at the same time increases with *λ*. Consequently, the probability that more than two neighboring nodes simultaneously choose the same RCTS channel increases with *λ*, which results in a decrease in *Cr* with *λ* (Figure 7(b)).

**Figure 8:**
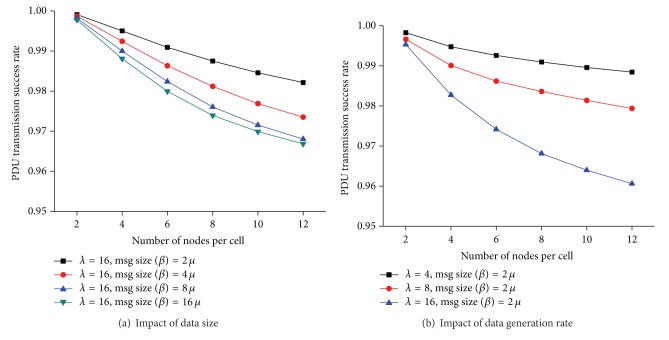
Data transmission success rate.

Figure 8 shows the influence of *ρ* on the success rate of data transmission. There are logical errors (LET4 and LET5) that cannot be detected through our distributed resource reservation protocol using only the 1-hop information of a node. When these undetectable logical errors occur, data transmission fails, even if a PDU bin is reserved successfully. Both the number of available PDU bins and the number of free RTS channels become smaller as the node density increases. Accordingly, it becomes more probable that neighboring nodes are attempting to reserve the same PDU bins using the same RTS channels at the same time. Consequently, since the LET4 and the LET5 take place more often with *ρ*, *Dr* decreases with the density of nodes, as shown in Figure 8.

Figures 8(a) and 8(b) show the impact of *μ* and *λ* on *Dr*. As the size of data increases, a node holds the reserved PDU bin longer. Thus, the level of contention for a PDU bin increases with the size of data. Similarly, the number of data a node needs to send per second grows as *λ* increases. This makes the contention levels for an RTS channel and a PDU bin increase. Accordingly, the probabilities that LET4 and LET5 take place become higher because it is more likely that nodes are reserving the same PDU bin at the same time. As a consequence, *Dr* decreases with the data size and the data generation rate. However, since *Dr* is more than 95% for all the simulation environments, the gain in our design choice when a node detects an LET3 outweighs the loss because of the LET5.

### 4.2. System Level Performance

In this section, we evaluate the performance of the proposed resource management method at the system level by considering not only the bit errors in a wireless link, but also the mobility of a node. In this work, we use a simplified packet error rate table shown in Table 2 to exclude the effects of physical layer issues of the resource management problem and focus on verifying the ability of our MAC protocol. When a receiver detects an error in a message, we assume that the receiver immediately discards the message without further processing.

We modify the random waypoint model [21] to represent the mobility pattern of a node. At the beginning of a simulation, a node uniformly selects the speed in [0, 72 km/h] and the direction in [0, 2*π*]. We assume that nodes do not change the initial speed and direction until the end of the simulation. If a node moves beyond the simulation topology, the node enters the network at a symmetrical point to the position where it moves out with respect to the center of the topology. We measure *Cr* and *Dr* by varying *λ*, *μ*, *T*, and *ρ*.

**Figure 9:**
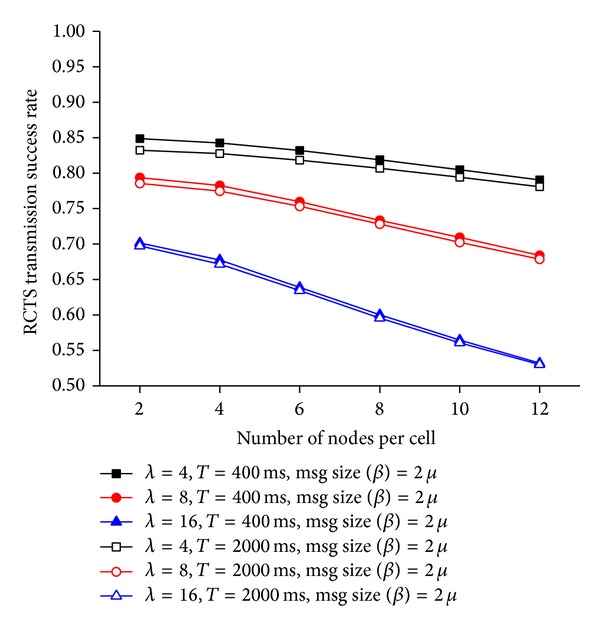
Resource reservation success rate in a mobile ad hoc environment (*λ* = 16).

The level of resource contention grows as the node density increases. Therefore, the contention success rate deteriorates with *ρ* (Figure 9). However, compared with that in the ideal simulation environment, *Cr* becomes lower in this simulation environment under the same *λ*, *μ*, *T*, and *ρ*. This is attributed to the frame loss and the mobility of a node that prevents the successful RTS/CTS frame exchange besides the physical errors. A node cannot reserve a PDU bin if an RTS frame or a CTS frame is lost in a wireless link. Furthermore, if one of the nodes performing a resource reservation process moves out of the transmission range of the other node, it cannot complete the RTS frame and the CTS frame exchange. In the range of the parameters, the data generation rate dominates *Cr* because it has the greatest effect on the frequency at which a node begins a resource reservation process.

**Figure 10:**
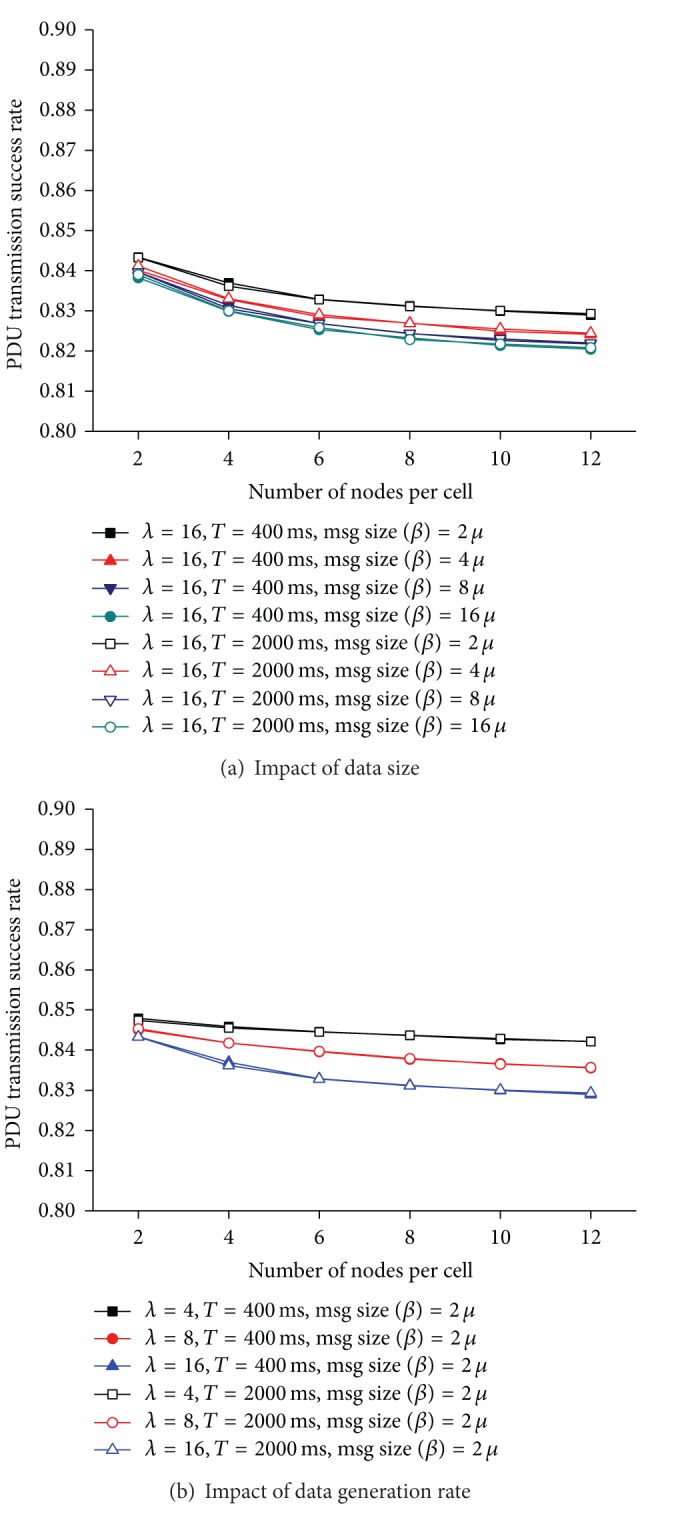
Data transmission success rate in a mobile ad hoc environment.

Figure 10 shows the success rate of data transmission. In addition to the loss in data transmission, the logical errors that could not be detected by local information also result in data transmission failure. Since a node manages *U*_*X*_ and *A*_*X*_ by overhearing messages not destined to it, *U*_*X*_ and *A*_*X*_ become more inaccurate as the number of lost frames increases. Therefore, the probability that a node reserves a PDU bin that is being used by its 1-hop or 2-hop neighbors grows. Consequently, the increase in the number of the LET4 and the LET5 reduces *Dr*. Moreover, since nodes are moving around, data transmission also fails if a node moves out of the transmission range of its corresponding node after successfully reserving a PDU bin. The larger the data size becomes, the longer the reserved PDU bin is used. If the number of available PDU bins becomes small, the LET5 is more likely to occur. Therefore, *Dr* deteriorates (Figure 10(a)) as the data size increases. However, since we limit the maximum number of data retransmissions to two, a PDU bin is returned if a data transmission does not succeed for two consecutive trials. Therefore, the decrease in *Dr* does not show significant difference with *μ*.

The parameter *T* controls the frequency at which a node announces its presence to a network. Thus, the time for a node to construct an exact NN_*X*_ decreases as *T* becomes smaller. If NN_*X*_ is equal to AN_*X*_, the NN_*X*_ can be considered to be exact. However, since a node maintains *U*_*X*_ not by NN_*X*_ but by the information in the ACK zone and uses NN_*X*_ to calculate the backoff probability when a collision occurs in a resource reservation process, the impact of *T* on the *Cr* and the *Dr* was marginal. On the other hand, the timeliness of an exact NN_*X*_ affects the performance of a routing protocol. Therefore, in the following section, we analyze the impact of *T* on the system performance in terms of the probability that a node successfully receives a NUM message from one of its neighbors. We also derive an optimal *T* that minimizes the average delay until a node successfully receives NUM messages from one of its neighbors.

### 4.3. Performance of Network Estimation Procedure

**Figure 11:**
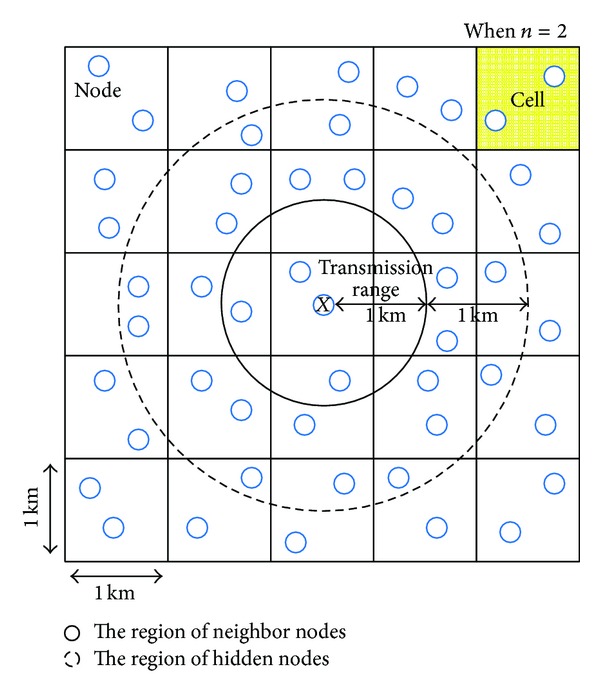
A simulation topology to evaluate the impact of  *T* (*n* = 2).

The simulation environment for the performance evaluation of the network estimation procedure is as follows. All the nodes are configured to have the same transmission radius of 1 km and the same broadcast period *T*. We uniformly deploy nodes in a 5 km × 5 km region. We denote the number of nodes per cell (i.e., the number of nodes in a 1 km × 1 km region) by *n*. To include the hidden nodes in the contention for the NMU channels, we select a node *X* randomly among the nodes located in the center cell of the topology (Figure 11). For the selected node *X*, *p*_NMU_ is calculated as the ratio of the number of nodes that successfully received the NMU message from *X* to |AN_*X*_| after *X* sends an NMU message. To evaluate the effect of  *T* on the performance of our protocol, we exclude the dynamics of a radio propagation environment by assuming that frames are not lost in a wireless link.

**Figure 12:**
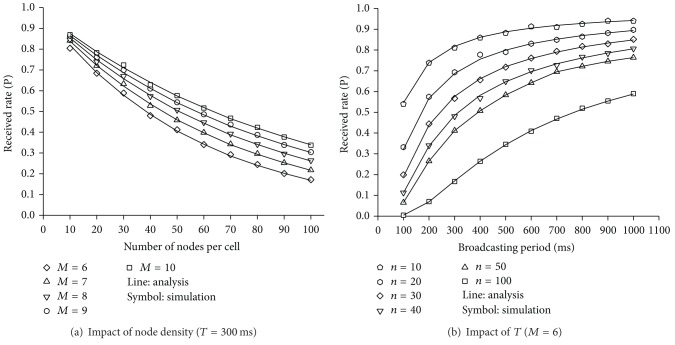
Probability of successfully receiving an NMU message.

As we can see in Figure 12, the *p*_NMU_ values obtained by the mathematical analysis are in accord with those from simulations for various node densities in a cell and *T*_*s*_. Given *T* and *M*, it is likely that nodes choose the same NMU channel at the same time as *n* increases. Therefore, *p*_NMU_ becomes smaller with the number of nodes per cell (Figure 12(a)). If a node density is given, the contention level for the NMU channels decreases as the number of NMU channels increases. Therefore, *p*_NMU_ becomes higher with *M*.  Figure 12(b) shows the impact of *T* on *p*_NMU_ for a few *n* when *M* = 6. As *T* becomes longer, it is more unlikely that more than two nodes select the same frame for advertising their presence. Thus, the probability of successfully receiving an NMU message increases with *T*.

**Figure 13:**
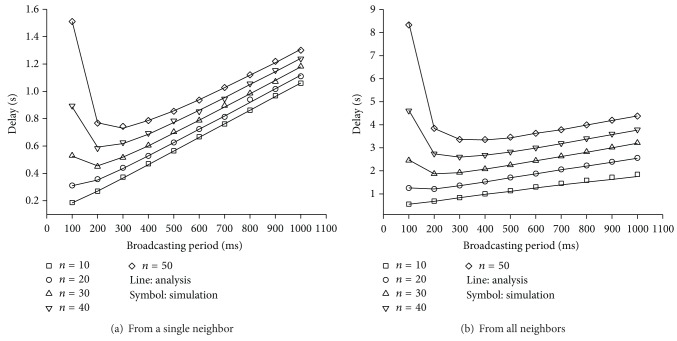
Average delay for a node to successfully receive the NMU message from its neighbors.

Figure 13(a) shows the average delay for a node to successfully receive an NMU message from one of its neighbors as *T* varies from 100 ms to 1000 ms when *M* = 6. The simulation results are in accord with the analytical results in (2). Since the contention level for an NMU channel decreases as the number of neighboring nodes becomes smaller, *D*_*o*_ becomes longer with *n*. For a given node density, we can see that there is an optimal *T* that minimizes *D*_*o*_. As can be seen in Figure 13(b), the changing pattern of *D*_*a*_ is the same as that of *D*_*o*_, and *D*_*a*_ from simulations also coincides with those from the analysis.

## 5. Conclusions and Future Works

We have proposed a distributed OFDMA-based MAC protocol for mobile ad hoc multihop networks. A MAC frame format that supports resource reservation before data transmission was presented. The proposed MAC frame format is divided into data and control subframes. The data subframe is composed of multiple PDU bins, each of which contains data to be transferred. The control subframe is composed of  NMU, ACK, and RCTS zones. The roles of each zone are explained in detail. Also, we classify logical error scenarios into 5 categories, from LET1 to LET5, some of which are unique features of the MAC protocol using OFDMA. By extensive simulation studies and analysis, we have evaluated the performance of the proposed MAC protocol and verified the effects of the distributed resource reservation procedure and inevitable logical error on system performance. Specifically, the RCTS/PDU transmission success rates were evaluated as functions of message size and traffic generation ratio in the MAC-level simulation results. Also, we have evaluated the system level simulation results of RCTS/PDU transmission efficiency, considering both bit errors in a wireless link and the mobility of a node. Finally, we analyzed and evaluated the effect of the NMU advertisement period on the delay before a node receives NMU messages from all of its neighbors.

In our work, a receiving node does not send ACK message when time-sensitive service is required by the upper layer. Instead, the receiving node can control the data transmission of the sending node by the feedback of data quality information such as the success rate of data transmission and data transmission delay. This transmission control methodology should consider the quality of service to be guaranteed in upper layers and optimized usage of system resources which will be the focus of further work.

## Figures and Tables

**Table 1:** The types of logical errors (*X*: a node requesting a reservation of a PDU bin and *Y*: a node receiving a resource reservation request from *X*).

	Cause	Detection method	Actions on detection
LET1	When *X* selects a PDU bin *i* for reservation, it could not know *U*_*Y*_.	Inspecting *U*_*Y*_.	*Y* sends a CTS frame with error type LET1. *X* restarts the reservation process.

LET2	Multiple node pairs attempt to reserve the same PDU bin using different RTS channels at the same time.	*Y* checks the RTS region of the frame from which it receives a resource reservation request.	*Y* sends a CTS frame with error type LET2. *X* restarts the reservation process.

LET3	Multiple nodes send RTS frames simultaneously to the same node *Y* through different RTS channels to reserve the same PDU bin.	*Y* checks the RTS region of the frame from which it receives resource reservation requests.	*Y* randomly selects a winner and assigns another PDU bin to a loser. If *X* is a loser, it uses the assigned PDU bin if it is available. If not, it restarts the reservation process.

**Table 2:** The characteristics of a wireless link in terms of delivering a message between a sender and a receiver. (Each column except the first one represents the probability that a message corresponding to its first row is received at a receiver without an error.)

Distance from a sender (km)	PDU	NMU	ACK	RTS	CTS
0.0–0.5	1	1	1	1	1
0.5–1.0	0.96	0.98	0.99	0.99	0.99
1.0–1.5	0.89	0.95	0.97	0.97	0.97
1.5–2.0	0.67	0.83	0.92	0.92	0.92
Otherwise	0	0	0	0	0

## Notations

NN_*X*_: An estimated set of 1-hop neighbor nodes of a node *X*
AN_*X*_: An actual set of 1-hop neighbor nodes of a node *X*
*U*_*X*_: An estimated set of PDU bins being used by 1-hop neighbors of a node *X* including those that *X* is using
*R*_*i*_^*j*^: An RTS frame sent through an RTS channel *j* to request to reserve a PDU bin *i*
*C*_*i*_^*j*^: A CTS frame sent through a CTS channel *j* to inform about the reservation state of a PDU bin *i*, which is requested by a node, starting a reservation process
*A*_*X*_: A set of PDU bins that 1-hop neighbors of *X* attempt to reserve when *X* receives an RTS frame from one of its neighbor nodes *Y*. *A*_*X*_ is inferred from the RTS region and it excludes the PDU bin that *Y* requests
*T*_*F*_: A frame length.
